# Bridging the Gap: Interventions to Increase Cancer Screening Adherence in Individuals with Mental Disorders—A Systematic Review

**DOI:** 10.3390/bs15010047

**Published:** 2025-01-04

**Authors:** Paolo Lombardo, Ilaria Mussetto, Valentina Baccolini, Enrico Di Rosa, Alessandra Sinopoli

**Affiliations:** 1 Department of Public Health and Infectious Disease, Sapienza University of Rome, Piazzale Aldo Moro 5, 00185 Rome, Italy; 2 Department of Prevention, Local Health Authority Roma 1, Borgo Santo Spirito, 3, 00193 Rome, Italy

**Keywords:** screening program, oncological screening, screening uptake, RCT, psychiatric, mental illness, screening behavior

## Abstract

Patients with mental illnesses adhere to organized cancer screening programs less frequently than the general population. This systematic review aims to examine the literature to identify studies that evaluate interventions designed to increase cancer screening adherence in people with mental disorders. The review protocol was registered (CRD42024510431) and Pubmed and Scopus were searched up to January 2024. Breast, colorectal, or cervical cancer screening were considered. We adhered to the PROSPERO guidelines. Study quality was assessed. Overall, six articles were included: two RCT studies, two before–after studies, and two cohort studies. Four interventions were conducted in the USA, one in Canada, and one in Japan. Two studies evaluated all three cancer screening programs, two studies evaluated breast cancer screenings, and two studies evaluated colorectal cancer screenings. The proposed interventions included patient navigation, case management, and support from staff members along with educational modules, decision counselling sessions, and enhanced primary care. The most consistent improvements in screening adherence were observed in breast and colorectal cancer screenings compared to usual care, particularly through interventions like patient navigation (colorectal cancer: 47.1% vs. 11.8%, *p* < 0.001) and case management. Further evaluations of interventions and their costs are still needed.

## 1. Introduction

Population-based cancer screening programs, which entail the systematic use of diagnostic tests in asymptomatic individuals, have proven their safety and cost-effectiveness in detecting breast, cervical, and colorectal cancer at an early stage, leading to reduced mortality and morbidity rates (Ratushnyak et al., 2019). The World Health Organization (World Health Organization [WHO], 2010) actively promotes and endorses participation in these cancer screening programs, as well as enabling participation and addressing health inequalities (World Health Organization, Regional Office for Europe, 2022).

Cancer screening is a process for detecting cancer in asymptomatic people at an early stage. Cancer screening is, therefore, considered to be a secondary prevention. Within each cancer screening program, there is a provision for different screening tests to be performed in succession in case of positivity from the least invasive to the most invasive. This is the case, for example, for colorectal cancer screening, where, generally, the stool test is performed first, followed, in case of positivity, by a colonoscopy. In an organized screening program, the health care provider directly invites the entire segment of the population deemed to be at risk of developing a certain disease, offering the test and any further investigations free of charge. Adherence to the program is entirely voluntary. To be planned and implemented, screening must address diseases of high epidemiological significance, be evidence-based, and follow quality guidelines. Screening programs have been shown to be effective in changing the natural history of breast, cervical, and colorectal cancers. Early detection still allows for minimally invasive and nondestructive interventions (Barratt, 2006). Cancer screening programs also present downsides. These include the psychological and physical harms of false positive and false negative tests, the potential to uncover clinically irrelevant diseases (pseudo-disease), and, therefore, harm from unnecessary treatment (EpiCentro, n.d.). The effectiveness of a cancer screening program strongly relies on its adherence rate (Baccolini et al., 2022). Unfortunately, several barriers to access to cancer screening programs among people with psychiatric pathologies are present (Prochaska et al., 2017; Tuschick et al., 2024; Zoorob et al., 2022). Several studies have highlighted a direct correlation between psychiatric conditions and a reduced participation in cancer screening programs (Harder et al., 2018; Kirkøen et al., 2023; Ross et al., 2021; Solmi et al., 2020). A meta-analysis involving over 4.7 million individuals has demonstrated how adherence to organized cancer screening programs is significantly less frequent in people with mental illnesses compared to the general population with respect to breast cancer, cervical cancer, and prostate cancer, but not for colorectal cancer (Solmi et al., 2020).

**Figure 1:**
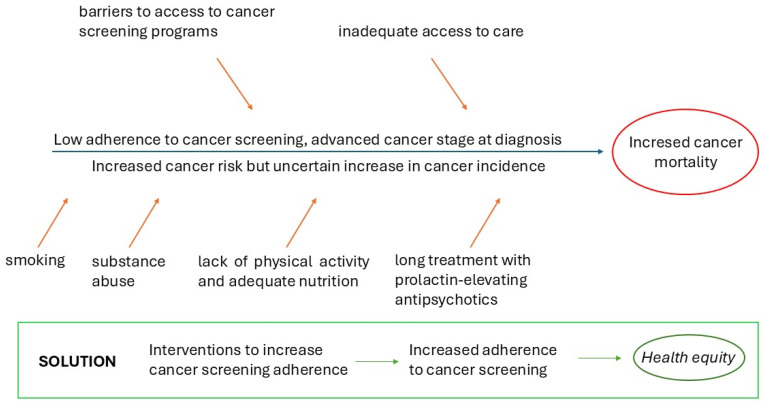
Fishbone diagram or Ishikawa diagram to synthesize issues surrounding cancer screening in people with mental illnesses and the solution to the problem.

People with a mental illness, therefore, require special consideration. Research shows that people with mental disorders experience an increased mortality rate from cancer compared to the general population (Howard et al., 2010; Kisely et al., 2008; Nordentoft et al., 2013), while data on cancer incidence are contrasting, with some studies suggesting an increased incidence of cancer in individuals with severe mental illnesses (Howard et al., 2010; McGinty et al., 2012) and others reporting a similar overall cancer incidence (Kisely et al., 2008, 2013) compared to the general population. However, the increased cancer risk in people with mental illnesses can also be attributed to the increased prevalence of unhealthy behaviors such as smoking (Prochaska et al., 2017), substance abuse (Sánchez Autet et al., 2018), lack of physical activity (Vancampfort et al., 2017), lack of adequate nutrition (Dipasquale et al., 2013), or long treatment with prolactin-elevating antipsychotics (Rahman et al., 2022). Conversely, excess mortality rates might reflect an advanced cancer stage at diagnosis (Céspedes et al., 2020; Davis et al., 2020) and inadequate access to care (Protani et al., 2022). Figure 1 summarizes these cause-and-effect relationships.

For these reasons, there is an urgent need to find effective strategies to improve adherence to cancer screening programs among patients with psychiatric disorders so that evidence-based interventions tailored specifically to this hard-to-reach population are identified and integrated into clinical practice. Previously, reviews have already been conducted on this issue, but they are outdated and have not yielded a sufficient volume of evidence (Barley et al., 2013, 2016; Lamontagne-Godwin et al., 2018; L. C. Weinstein et al., 2016). Therefore, our systematic review aims to re-examine the literature to identify interventions to increase cancer screening adherence in people with mental disorders. We intend to provide a synthesis of the results to support public health policy to reduce disparities in cancer screening participation rates.

## 2. Methods

This systematic review adhered to the guidelines outlined in the Cochrane Handbook for Systematic Reviews and the Preferred Reporting Items for Systematic Reviews and Meta-Analyses statement (*Cochrane handbook for systematic reviews of interventions*, n.d.; Moher et al., 2009). The review protocol was registered on PROSPERO under the identifier CRD42024510431. Since the study did not involve primary data collection, it was not required to obtain institutional review board approval or informed consent.

### 2.1. Inclusion Criteria

Experimental studies, such as randomized–controlled trials (RCTs) or before–after studies, and observational studies, like cohort studies, that report data on interventions aimed at increasing adherence to cancer screening programs in people with mental illnesses were considered eligible. Specifically, we included studies: (i) reported in English, Italian, Spanish, or Portuguese, based on the co-authors’ language abilities, (ii) conducted in any country, (iii) that quantify adherence to colorectal, breast, or cervical cancer screening programs, and (iv) that consider, as the target population, people with any type of mental disorder (e.g., schizophrenia, depression, bipolar disorder). We excluded review articles, letters to the editor, conference papers, or any other article in which data on interventions to increase adherence to cancer screening programs in people with mental illnesses were not retrievable.

### 2.2. Search Strategy

PubMed and Scopus, because of their relevance in the medical field and the experience gained by researchers, were searched to retrieve potentially eligible articles published from the inception until January 2024. The search terms were related to mental diseases and cancer screening, such as “mental illness”, “oncological screening”, “intervention”, and synonyms. There was no language or date restriction. The string was adapted to fit both databases’ research criteria (Supplementary Table S1).

Duplicate articles were removed using the Zotero software, and the title and abstract of the collected records were screened independently by two reviewers (P.L., I.M). Studies that clearly did not meet the inclusion criteria were excluded. Two researchers retrieved the full texts of potentially relevant articles (P.L., I.M.). Disagreements were resolved through discussion with a third reviewer (V.B., A.S.), and the reasons for exclusion were recorded. Additionally, the literature search was supplemented by scanning the reference lists of the articles retrieved and by searching ClinalTrials.gov for additional records.

### 2.3. Data Collection, Synthesis, Study Quality

For each eligible study retrieved, two reviewers (P.L., I.M.) extracted the following information: first author, year of publication, study design, country, sample size, characteristics of the target population (type of mental illness, number of participants, age range, gender), recruitment setting, type of cancer screening (i.e., colorectal, breast, or cervical), type of intervention, follow-up time, and outcome measures expressed as a change in the uptake of cancer screening programs (raw data, percentage, or odds ratio—ORs—and 95% confidence interval—CI). A.S. supervised the data extraction.

Two authors (P.L., I.M.) independently performed the quality assessment of the articles included using the Cochrane risk-of-bias version 2 tool for randomized trials, the ROBINS-I tool for non-randomized studies of interventions, and the Newcastle Ottawa Scale for cohort studies (Tables S3 and S4). With respect to the latter, articles were considered to be of good quality when the total score was ≥7, fair quality if the score was ≥5 and <7, and poor quality if the score was lower than 5. Discrepancies were resolved by achieving consensus. Articles were then grouped according to the study design, and the results were narratively synthesized.

## 3. Results

**Figure 2:**
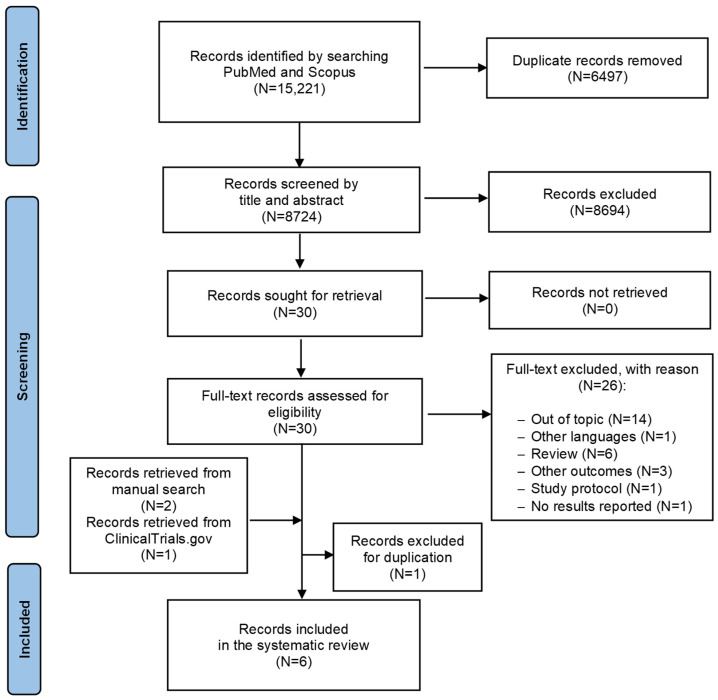
PRISMA flow diagram of the review process.

The search identified 15,221 records (Figure 2). A total of 30 reports were considered to be eligible for full-text analysis. Two records were added to the previous four from the reference lists of relevant articles, and one record retrieved from ClinicalTrials.gov met our inclusion criteria. However, the latter was excluded because the publication had already been included, leading to six articles ultimately being included in the systematic review.

### 3.1. Characteristics of the Included Studies

The six included studies (Abuelo et al., 2020; Fujiwara et al., 2021; Grove et al., 2021; Heyding et al., 2005; Murphy et al., 2020; L. Weinstein et al., 2019) were published between 2005 and 2021 (Table 1). Regarding the study design, two studies were RCTs (Abuelo et al., 2020; Fujiwara et al., 2021), two were before–after studies (Heyding et al., 2005; L. Weinstein et al., 2019), and two were observational studies (Grove et al., 2021; Murphy et al., 2020) (i.e., cohort studies). Most studies were conducted in North America (*n* = 5), especially in the United States of America (*n* = 4). Only one study was conducted in Asia, i.e., Japan (Fujiwara et al., 2021). Most of the studies assessed individuals with severe mental illnesses (*n* = 4), two of which focusing on individuals with schizophrenia or schizoaffective disorders (Fujiwara et al., 2021; Grove et al., 2021). Four studies investigated patients of both sexes (Abuelo et al., 2020; Fujiwara et al., 2021; Grove et al., 2021; Murphy et al., 2020), whereas the remaining two studies focused on women only (Heyding et al., 2005; L. Weinstein et al., 2019). Two studies assessed adherence to all three cancer screening programs (Fujiwara et al., 2021; Murphy et al., 2020), two focused on breast cancer screening (Heyding et al., 2005; L. Weinstein et al., 2019), and two on colorectal cancer screening (Abuelo et al., 2020; Grove et al., 2021). Almost all of the studies were conducted in a community setting (Abuelo et al., 2020; Grove et al., 2021; Heyding et al., 2005; Murphy et al., 2020; L. Weinstein et al., 2019), such as primary care or supportive housing (L. Weinstein et al., 2019). Only one study was conducted in psychiatric hospital outpatient clinics (Fujiwara et al., 2021).

As for the quality assessment, the two RCTs were judged to have a low risk of bias, with the main issues being the changing of the screening test after the study had already begun (Abuelo et al., 2020) and the effect of adhering to the intervention (Fujiwara et al., 2021). The overall bias risk in the non-randomized studies was deemed to be moderate, with the main issues being the bias in the selection of the participants to the studies. The study quality of the cohort studies was good (Supplementary Table S2). Nonetheless, these biases had no impact on how the results were interpreted.

### 3.2. Main Findings by Study Design

#### 3.2.1. RCT

The two randomized clinical trials enrolled a population inclusive of both sexes and compared adherence to cancer screening programs between individuals undergoing the intervention under study and usual care, with follow-up periods of six and nine months, respectively (Abuelo et al., 2020; Fujiwara et al., 2021) (Table 2). Sample sizes were similar, but, while Abuelo et al. considered only colorectal screening in a primary care setting, Fujiwara et al. considered all three cancer screening programs in a hospital setting (Abuelo et al., 2020; Fujiwara et al., 2021). The proposed intervention by Abuelo et al. involved a program in which navigators provide guidance, education, and address patients’ obstacles through either phone calls or face-to-face interactions and assist them during the testing process when required (Abuelo et al., 2020) (Table 3). On the other hand, Fujiwara et al. proposed an approach based on case management with three counseling sessions conducted by either a nurse or a psychiatric social worker to support and assist the participants by resolving issues or by motivating them to engage with cancer screening programs (Fujiwara et al., 2021) (Table 3). People with a single mental disease and with multiple mental diseases were included in the sample that Abuelo et al. investigated (Abuelo et al., 2020). Both studies observed a significantly higher proportion of adherence to colorectal cancer screening in the population that received the intervention compared to the control group (19.8% vs. 10.4%, *p* = 0.04; and 47.1% vs. 11.8%, *p* < 0.001, respectively) (Abuelo et al., 2020; Fujiwara et al., 2021). However, Fujiwara and colleagues did not observe a significant difference in cancer screening uptake in relation to cervical and breast cancer screening programs (*p* = 0.137 and *p* = 0.106, respectively) (Fujiwara et al., 2021) (Table 4).

#### 3.2.2. Before—After Studies

Both before–after studies quantified adherence to breast cancer screening using mammography as a test in female individuals, with follow-up periods of 12 and six months, respectively (Heyding et al., 2005; L. Weinstein et al., 2019) (Table 2). One intervention (Heyding et al., 2005) entailed a staff member accompanying women to their mammography appointments (Table 3), the other intervention (L. Weinstein et al., 2019), conducted on a small sample of women who had not undergone screening mammography in the past year, comprised an educational module and a decision counseling session supplemented with patient navigation services (Table 3). The first study demonstrated a significantly higher proportion of women undergoing mammography among those who received the intervention compared to those who did not (Heyding et al., 2005). In the second one, all the women included in the study received the intervention, and the participation rate reached 67% after the intervention (L. Weinstein et al., 2019). Moreover, while the first study (Heyding et al., 2005) was conducted in a primary care setting, the second was conducted in a supportive housing environment (L. Weinstein et al., 2019) (Table 4).

#### 3.2.3. Cohort Studies

The two cohort studies examined individuals of both sexes with severe mental illnesses and both were conducted in a primary care setting (Grove et al., 2021; Murphy et al., 2020) (Table 2). One study focused solely on colorectal cancer screening (Grove et al., 2021) among approximately 1600 individuals, while the other examined all three cancer screening programs in a large sample size (around 12,000 people) (Murphy et al., 2020). The intervention studied by Grove and colleagues aimed at enhancing primary care and regular communication with the patient’s behavioral health providers (Grove et al., 2021) (Table 3), while Murphy et al. focused on behavioral health homes integrating general medical services into specialty mental health settings (Murphy et al., 2020) (Table 3). The first study found a higher adherence rate to colorectal cancer screening in the intervention group compared to the control group. Still, it did not reach statistical significance (21% vs. 11%, *p* = 0.063) (Grove et al., 2021). In contrast, the second study showed a positive association between receiving the intervention and adherence to cervical and breast cancer screening (OR: 1.20, 95% CI: 1.07–1.35, *p* = 0.002; OR: 1.30, 95% CI: 1.06–1.59, *p* = 0.01, respectively), but not for colorectal cancer screening (OR: 0.97, 95% CI: 0.82–1.13, *p* = 0.66) (Murphy et al., 2020).

## 4. Discussion

Differences in the participation in screening programs between the population with psychiatric diseases and the rest of the population have prompted the scientific community to test new strategies that may allow for the problem of reduced adherence to be mitigated. However, few experimental studies on the subject have emerged regarding the urgency of finding solutions for this particularly exposed population, despite issues acknowledged in scientific literature such as barriers to cancer screening attendance (Tuschick et al., 2024), double the likelihood of dying from cancer under the age of 75 (*Severe Mental Illness [SMI]: Inequalities in Cancer Screening Uptake Report*, 2021, September 21), prevalence of unhealthy behaviors (Dipasquale et al., 2013; Prochaska et al., 2017; Sánchez Autet et al., 2018; Vancampfort et al., 2017), or long treatment with prolactin-elevating antipsychotics (Rahman et al., 2022).

Our research identified three different types of studies with both experimental and observational designs: RCT, before–after, and cohort studies. The main care setting studied was primary care, except for two studies where the hospital setting was the one studied (Fujiwara et al., 2021; Heyding et al., 2005). The target population included in the studies was almost comparable: in most of the studies, the subjects involved suffered from psychiatric pathologies identifiable as Severe Mental Illness (SMI).

Weinstein et al. (L. Weinstein et al., 2019), Heyding et al. (Heyding et al., 2005), and Abuelo et al. (Abuelo et al., 2020) achieved good results in terms of cancer screening adherence following the intervention. These three studies are related by the presence of an additional operator, either the navigator or a staff member who plays a similar role. The navigator engaged with each patient individually, and it can be argued that this tailored approach to patient interaction helps lower barriers to the access of care. The intervention proposed by Weinstein et al. also includes educational and decision-support sessions (L. Weinstein et al., 2019). However, the high percentage of adherence obtained in this study should be viewed differently from the other works. In fact, unlike the other studies that included a comparison group, this study’s population consisted of women with SMI who had not undergone a screening mammography in the past year, and all of them received the intervention. Abuelo et al. (Abuelo et al., 2020) highlights that improving the feasibility of the intervention requires proper training for the navigators and more time for them to dedicate to the activities. Similarly, Weinstein et al. (L. Weinstein et al., 2019) suggests enhancing the intervention through better staff training and improved communication between the medical and the housing teams.

Fujiwara’s et al. work also showed good results in colorectal cancer screening adherence by using a case manager figure whose tasks were closely aligned with those of a navigator (Fujiwara et al., 2021). The retrospective studies by Grove et al. and Murphy et al., on the other hand, evaluated the outcomes of services providing care through different approaches in real-world settings, specifically assessing the enhanced primary care model in patient-centered medical homes and the behavioral health home model, respectively (Grove et al., 2021; Murphy et al., 2020). Murphy et al. obtained promising results (Murphy et al., 2020).

The implementation of these interventions in different contexts may be constrained by various barriers, such as limited resources, the need for adequately trained personnel, and the specific structure and organization of healthcare systems. Therefore, further research is essential to assess how these interventions can be adapted and effectively implemented across a diverse range of healthcare settings.

The problem of unsatisfactory adherence to organized cancer screening programs, unfortunately, affects a broad category of people often defined as fragile, such as ethnic minorities. A systematic review conducted on interventions to improve adherence to cervical screening among ethnic minorities demonstrates that telephone support with navigation is effective with moderately strong evidence (Glick et al., 2012). Another systematic review demonstrates the effectiveness of patient navigator interventions and educational workshops (Mohamed et al., 2024). Therefore, even though the frail population is different, treatments that rely on the presence of an intermediary facilitator to access care have been shown to be a key common resource when applied.

There have been previous attempts to gather knowledge from the literature on this subject. The two previous systematic reviews published in the Cochrane Database of Systematic Reviews in 2013 and 2016, respectively, titled “Interventions to encourage uptake of cancer screening for people with severe mental illness”, which included only RCTs, did not yield results (Barley et al., 2013, 2016). Another realistic review (Lamontagne-Godwin et al., 2018) found two articles on breast cancer screening (Heyding et al., 2005; Xiong et al., 2015). However, one of them did not meet our inclusion criteria, being a cross-sectional study comparing three different clinical settings (Xiong et al., 2015).

Scientific studies propose and examine targeted interventions to improve screening adherence in other hard-to-reach vulnerable populations, such as migrants (Mohamed et al., 2024) and people experiencing homelessness (Baggett et al., 2024). A recent systematic review has found that, among migrant populations, home HPV tests, educational workshops for women, and training for general practitioners increased cervical cancer screening adherence, while patient navigator interventions improved breast cancer screening rates (Mohamed et al., 2024).

The strengths of our systematic review include the diverse range of study types, encompassing RCTs, before–after studies, and cohort studies, as well as the evaluation of interventions in both experimental and observational settings. Additionally, it provides a comprehensive assessment of all three types of cancer screening—breast, cervix, and colorectal—and benefits from the recruitment of populations from various settings. However, our review also has various weaknesses: the heterogeneity of the included study types and the small sample size. Out of the six studies collected, four were conducted in the U.S., and this makes the results narrow in the global context and potentially unsuitable for generalization to nations with limited resources. Although we observed that the primary care context produces very good results, another limitation is that most of the research is conducted in this setting, meaning that generalizations regarding which care setting is associated with the most beneficial interventions cannot be made. However, the reason for this can be as simple as the fact that such environments are the ones in which people with psychiatric disorders are reached in common clinical practice.

There is an exclusion of results from different cancer screenings, like lung and gastric cancers. Additionally, there is a focus primarily on severe mental illnesses which may limit the inclusion of other less severe but more common psychiatric conditions. Overall, the quality of the studies included was satisfactory.

Analyzing the costs of interventions is necessary to ensure that those providing the best health outcomes at the lowest cost are implemented, thereby optimizing resource use and effectiveness. Doing this is complex because it requires a detailed breakdown of each cost item. The calculated cost should then be compared to the health outcomes obtained. Unfortunately, none of the studies report a detailed cost analysis. However, considerations were made regarding the extra time devoted by the staff to the intervention. Specifically, in the Abuelo et al. study, the staff had to allocate a portion of their time, equivalent to 0.15 of full-time work (Abuelo et al., 2020), to the intervention, while, in the Fujiwara study, the initial in-person counseling lasted an average of 13 min (Fujiwara et al., 2021). Future studies are needed to evaluate not only the effectiveness of the interventions, but also their costs; therefore, a cost-effectiveness analysis is needed. Other potential resource requirements, regardless of their strictly economic nature, that could reduce the feasibility of the interventions are staff training and education, infrastructure adaptation, the purchase of new equipment and devices, and the reorganization of existing services. We believe that both clinical trials with diverse and representative samples conducted in research settings and observational studies in real-world environments are essential for identifying the most effective interventions for implementation. These evaluations should be conducted mainly in primary care settings to achieve health equity (Richard et al., 2016).

## 5. Conclusions

Interventions targeted at people with mental illnesses have often demonstrated increased cancer screening adherence with respect to all three cancer screening programs.

The following interventions were found to increase adherence to breast and colorectal cancer screening: case management and patient navigation. Additionally, educational modules, decision counseling, and being accompanied by a staff member to the visit were effective for breast cancer screening. Behavioral health homes were found to improve screening rates for cervical cancer. Most of the identified interventions were conducted in primary healthcare settings with a high rate of success.

Interventions that were more effective than standard care involved the use of additional personnel, entailing the allocation of extra resources. There is a clear need for new studies to assess the effectiveness of specific interventions among people with mental illnesses and to also focus on the issue of the resulting increase in cost.

Health policies should insist on bridging the gap in cancer screening disparities between people with mental illnesses and the general population by investing more resources in primary health settings.

## Figures and Tables

**Table 1:** Study sorting based on study type. Key characteristics of the studies included in the systematic review by study design.

First Author, Year	Study Design	Country	Target Population	Cancer Screening Program	Recruitment Setting	Risk of Bias/Overall Bias/Study Quality
(Abuelo et al., 2020)	RCT	USA	Patients with mental illnesses and/or substance use disorders	Colorectal cancer	Healthcare center	Some concerns
(Fujiwara et al., 2021)	RCT	Japan	Patients with schizophrenia or schizoaffective disorders	Colorectal, breast, and cervical cancer	Hospital psychiatry outpatient clinics	Some concerns
(Heyding et al., 2005)	Before–after	Canada	Women with any psychiatric or substance abuse diagnosis	Breast cancer	Inner-city drop-in center	Moderate risk
(L. Weinstein et al., 2019)	Before–after	USA	Women with serious mental illnesses who had not undergone a mammography in the last year	Breast cancer	Supportive housing programs	Moderate risk
(Grove et al., 2021)	Cohort	USA	Patients with schizophrenia or schizoaffective disorders	Colorectal cancer	Primary care	Good quality
(Murphy et al., 2020)	Cohort	USA	Patients with serious mental illnesses	Colorectal, breast, and cervical cancer	Psychiatric rehabilitation programs	Good quality

RCT: Randomized Controlled Trial. USA: United States of America.

**Table 2:** Study sorting based on study type. Principal effects of interventions aimed at individuals with mental illnesses.

Author, Year	Study Design	Setting	Intervention and Control/Comparison	Age *, Sex	Sample Size (N)	Screening Test	Follow-Up	Results ^#^ (Intervention vs. Control, or Post- vs. Pre-Intervention, or Treatment vs. Comparison Group)
(Abuelo et al., 2020)	RCT	Primary care	-Patient navigation program -Usual care	50–74 years, both sexes	Intervention group: 126, control group: 125	FOBT or CS	6 months	Colorectal cancer: 19.8% vs. 10.4%, *p* = 0.04
(Fujiwara et al., 2021)	RCT	Hospital outpatient clinic	-Case management -Usual recommendations provided by the municipality	39–80 years, both sexes	Intervention group: 86, control group: 86	FOBT, MG, and PAP test	9 months	Colorectal cancer: 47.1% vs. 11.8%, *p* < 0.001 Breast cancer: 14.3% vs. 4.8%, *p* = 0.137 Cervical cancer: 19.1% vs. 7.1%, *p* = 0.106
(Heyding et al., 2005)	Before–after	Primary care	-Being accompanied by a known and trusted staff member -Routine primary care and cancer screening at drop-in centers	50–70 years, females	Pre-intervention: 158, post-intervention: 89	MG	1 year	Breast cancer: 29.2% vs. 4.7%, *p* = 0.001
(L. Weinstein et al., 2019)	Before–after	Supportive house	Educational module, decision counseling session, and patient navigation	53.2 (5.6) years, females	Pre-intervention: 21, post-intervention: 21	MG	6 months	Breast cancer: 67.0% vs. 0.0%
(Grove et al., 2021)	Cohort	Primary care	-Enhanced primary care -Usual primary care	50–75 years, both sexes	Treatment group: 160, comparison group: 1433	NS	18 months	Colorectal cancer: 21% vs. 11%, *p* = 0.063
(Murphy et al., 2020)	Cohort	Primary care	-Behavioral health homes -Usual care	21–64 years, both sexes	Treatment group: 3298, comparison group: 8878	FOBT, SS, CS, MG, and PAP test	3 years	Colorectal cancer: OR: 0.97 (0.82–1.13), *p* = 0.66; breast cancer: OR: 1.30 (1.06–1.59), *p* = 0.01; cervical cancer: OR: 1.20 (1.07–1.35), *p* = 0.002

APSS: Adult Psychiatric Support Services. CRC: Colorectal Cancer. CS: Colonoscopy. FOBT: Fecal Occult Blood Test. MG: Mammography. NA: Not Applicable. PAP: Papanicolaou. PCPs: Primary Care Physicians. SS: Sigmoidoscopy. NS: Not Specified. * age is expressed as the range or the mean (standard deviation). ^#^ results are expressed as the screening uptake proportion or odds ratio (OR) and its associated 95% confidence interval.

**Table 3:** Study sorting based on study type. Key components of the proposed interventions and the estimated additional amount of workforce needed.

Author, Year	Study Design	Type of Intervention	Intervention	Usual Care	Additional Workforce Required
(Abuelo et al., 2020)	RCT	Patient navigation program	Through at least one telephone or in-person conversation, navigators educated patients about colorectal cancer and screening, explored their barriers, reminded patients about the test, helped with translation or cultural issues, and resolved insurance matters. The navigators accompanied patients to the test when necessary.	CRC screening as arranged by the primary care team	Navigators
(Fujiwara et al., 2021)	RCT	Case management	Three counseling sessions. The first in-person session included: (a) education about the importance and content of colorectal cancer screening, using a pamphlet, (b) assistance in making decisions and an appointment for colorectal cancer screening, and (c) assistance in obtaining a coupon for free cancer screening, if necessary. Participants received at least two follow-up counseling sessions (in-person or over the telephone), support and encouragement to participate, and adjustment of follow-up contacts based on the clinical assessment.	Usual recommendations provided by the municipality	Case manager (nurse or psychiatric social worker)
(Heyding et al., 2005)	Before–after	Being accompanied by a known and trusted staff member	A staff member contacted and accompanied small groups of women to their mammography visits, at a pre-arranged time.	Routine primary care services and breast and cervical cancer screening at drop-in centers	More working hours required from a staff member
(L. Weinstein et al., 2019)	Before–after	Mammography decision support and patient navigation intervention	Patients underwent an educational module and a decision counseling/support session, followed by tailored navigation. In the navigation component, patients were contacted monthly by phone or in-person to receive support for their decisions and assistance with scheduling mammograms, obtaining referrals, arranging transportation, and following up on abnormalities if needed.	N/A	Educators, staff for decision counseling and navigation
(Grove et al., 2021)	Cohort	Enhanced primary care in a patient-centered medical home	Individuals received an enhanced primary care visit. Enhanced primary care included small patient panels, allowing providers to spend more time with each patient, training for providers, and regular communication between PCPs and the patients’ behavioral health providers to address the patients’ complex needs. PCPs addressed the patients’ health needs in collaboration with other medical providers.	Usual primary care visit	The need for additional workforce with specific tasks is not made directly explicit
(Murphy et al., 2020)	Cohort	Behavioral Health Homes (BHH)	BHH model: general medical services integrated into specialty mental health settings.	No enrollment in BHH	The need for additional workforce with specific tasks is not made directly explicit

RCT: Randomized Controlled Trial. CRC: Colorectal Cancer. NA: Not Applicable.

**Table 4:** Assessment of the efficacy of the interventions by type of cancer and care setting.

		Statistically Significant Increase Due to the Intervention	Non-Statistically Significant Increase	Ratio of Effective to Ineffective Interventions
Cancer Type	Colorectal	(Abuelo et al., 2020; Fujiwara et al., 2021)	(Grove et al., 2021; Murphy et al., 2020)	2:2
	Cervical	(Murphy et al., 2020)	(Fujiwara et al., 2021)	1:1
	Breast	(Heyding et al., 2005; L. Weinstein et al., 2019 *; Murphy et al., 2020)	(Fujiwara et al., 2021)	3:1
Clinical Setting	Primary care	Colorectal (Abuelo et al., 2020); breast (Heyding et al., 2005); breast (Murphy et al., 2020); cervical (Murphy et al., 2020)	Colorectal (Grove et al., 2021); colorectal (Murphy et al., 2020)	4:2
	Hospital outpatient clinic	Colorectal (Fujiwara et al., 2021)	Breast (Fujiwara et al., 2021); cervical (Fujiwara et al., 2021)	1:2
	Supportive house	Breast (L. Weinstein et al., 2019) *		1:0

* *p*-value not available.

## Supplementary Materials

The following supporting information can be downloaded at: https://www.mdpi.com/article/10.3390/bs15010047/s1, Table S1: Search strategies used in the systematic review; Table S2: Quality assessment of the articles included in the systematic review according to the Cochrane risk-of-bias version 2 tool for randomized trials; Table S3: Quality assessment of the articles included in the systematic review according to the ROBINS-I tool for non-randomized studies of interventions; Table S4: Quality assessment of the articles included in the systematic review according to the Newcastle Ottawa Scale for cohort studies.

## List of Abbreviations

WHO	World Health Organization
RCT	Randomized Controlled Trials
CRC	Colorectal Cancer Screening
SMI	Severe Mental Illness
BHH	Behavioral Health Homes
