# Supplementary material for: Bridging the Gap: Interventions to Increase Cancer Screening Adherence in Individuals with Mental Disorders—A Systematic Review
Source: Behav Sci (Basel). 2025 Jan 4;15(1):47. doi: 10.3390/bs15010047 (PMC11761912; doi:10.3390/bs15010047)
Supplement: Supplementary file 1 [file behavsci-15-00047-s001.zip › behavsci-3266139-supplementary.pdf]

Supplementary Materials

Supplementary Table S1. Search strategies used in the systematic review.

Database	Search Strategy	N
PubMed	“Substance use disorder*” OR “Mental illness*” OR “mental” OR “Psychiatric disease*” OR SUD OR “mental disorder*” OR “mental disease*” OR “mental sickness” OR SMI AND “Oncological screening” OR “secondary prevention” OR “cancer screening” OR “mass screening” OR “organized screening program*” OR “tumor screening” OR “cancer prevention” OR “mammography” OR ”faecal-occult-blood” OR “pap-test” OR “HPV-test” OR “HPV test” OR “pap test” OR “cervical cancer” OR “colon cancer” OR “Breast cancer” OR “colonoscopy” OR “colorectal cancer” AND intervention* OR invitation* OR reminder* OR education OR counselling OR “mobile phone*” OR email* OR “improve access” OR “improve adherence” OR “improve compliance” OR “procedural changes” OR “increase acceptability” OR incentive* OR procedure*	6241
Scopus	TITLE-ABS-KEY (“Substance use disorder*” OR “Mental illness*” OR “mental” OR “Psychiatric disease*” OR SUD OR “mental disorder*” OR “mental disease*” OR “mental sickness” OR SMI AND TITLE-ABS-KEY (“Oncological screening” OR “secondary prevention” OR “cancer screening” OR “mass screening” OR “organized screening program*” OR “tumor screening” OR “cancer prevention” OR “mammography” OR ”faecal-occult-blood” OR “pap-test” OR “HPV-test” OR “HPV test” OR “pap test” OR “cervical cancer” OR “colon cancer” OR “Breast cancer” OR “colonoscopy” OR “colorectal cancer”) AND TITLE-ABS-KEY (intervention* OR invitation* OR reminder* OR education OR counselling OR “mobile phone*” OR email* OR “improve access” OR “improve adherence” OR “improve compliance” OR “procedural changes” OR “increase acceptability” OR incentive* OR procedure*)	8980

Methods for quality assessment

By examining the full text of the articles, the authors evaluated how well each item on the grading scale was fulfilled. For any non-uniformity in the final score of the rating scale and each individual item, the authors compared orally until rating uniformity was achieved. A third reviewer's involvement was not required.

Supplementary Table S2. Quality assessment of the articles included in the systematic review according to the Cochrane risk-of-bias version 2 tool for randomized trials.

Revised Cochrane risk-of-bias tool for randomized trials (RoB2)							
	**DOMAIN 1**	**DOMAIN 2**		**DOMAIN 3**	**DOMAIN 4**	**DOMAIN 5**	
**Author, year**	Risk of bias arising from the randomization process	Risk of bias due to deviations from the intended interventions (effect of assignment to intervention)	Risk of bias due to deviations from the intended interventions (effect of adhering to intervention)	Missing outcome data	Risk of bias in measurement of the outcome	Risk of bias in selection of the reported result	Overall risk of bias
Abuelo, 2020	Some concerns	Some concerns	Low	Low	Low	Low	Some concerns
Fujiwara, 2021	Low	Some concerns	Some concerns	Low	Low	Low	Some concerns

Supplementary Table S3. Quality assessment of the articles included in the systematic review according to the ROBINS-I tool for non-randomized studies of interventions.

Risk of Bias In Non-randomized Studies of Interventions (ROBINS-I) tool								
**Author, year**	**Bias due to confounding**	**Bias in selection of participants into the study**	**Bias in classification of interventions**	**Bias due to deviations from the intended interventions**	**Bias due to missing data**	**Bias in measurement of outcomes**	**Bias in selection of reported result**	Overall risk of bias
Heyding, 2005	Moderate	Moderate	Low	Low	Low	Low	Low	Moderate risk
Weinstein, 2019	Low	Moderate	Low	Low	Low	Moderate	Low	Moderate risk

Supplementary Table S4. Quality assessment of the articles included in the systematic review according to the Newcastle Ottawa Scale for cohort studies.

**Newcastle Ottawa Scale for cohort studies**										
Author, year	Selection				Comparability		Outcome			Overall
Grove, 2021	1	1	1	1	1	0	1	1	1	8
Murphy, 2020	1	1	1	1	1	0	1	1	0	7
